# Pathogenetic basis of Takenouchi-Kosaki syndrome: Electron microscopy study using platelets in patients and functional studies in a *Caenorhabditis elegans* model

**DOI:** 10.1038/s41598-019-40988-7

**Published:** 2019-03-14

**Authors:** Tomoko Uehara, Hidenori Suzuki, Nobuhiko Okamoto, Tatsuro Kondoh, Ayesha Ahmad, Bridget C. O’Connor, Sawako Yoshina, Shohei Mitani, Kenjiro Kosaki, Toshiki Takenouchi

**Affiliations:** 1 Center for Medical Genetics, Keio University School of Medicine, Tokyo, Japan; 2 Division of Morphological and Biomolecular Research, Graduate School of Medicine, Nippon Medical School, Tokyo, Japan; 3 Department of Medical Genetics, Osaka Women’s and Children’s Hospital, Osaka, Japan; 4 Misakaenosono Mutsumi Developmental, Medical, and Welfare Center, Isahaya, Japan; 5 Division of Pediatric Genetics, Metabolism and Genomic Medicine, Department of Pediatrics, University of Michigan, Ann Arbor, MI USA; 6 Department of Physiology, Tokyo Women’s Medical University School of Medicine, Tokyo, Japan; 7 Department of Pediatrics, Keio University School of Medicine, Tokyo, Japan

## Abstract

The combined phenotype of thrombocytopenia accompanied by intellectual disability in patients with a *de novo* heterozygous mutation, i.e., p.Tyr64Cys in *CDC42*, signifies a clinically recognizable novel syndrome that has been eponymized as “Takenouchi-Kosaki syndrome” (OMIM #616737). In the present study, a detailed phenotypic analysis performed for a total of five patients with Takenouchi-Kosaki syndrome revealed that intellectual disability, macrothrombocytopenia, camptodactyly, structural brain abnormalities with sensorineural deafness, hypothyroidism, and frequent infections comprise the cardinal features of this condition. A morphologic analysis of platelets derived from three affected individuals was performed using electron microscopy. The platelets of the three patients were large and spherical in shape. Furthermore, platelet α-granules were decreased, while vacuoles were increased. We further performed a functional analysis of p.Tyr64Cys in *CDC42* through CRISPR/Cas9-mediated gene editing in a *Caenorhabditis elegans* model. This functional analysis suggested that the mutant allele has hypomorphic effects. Takenouchi-Kosaki syndrome is clinically recognizable by the combined phenotype of intellectual disability, macrothrombocytopenia, camptodactyly, structural brain abnormalities with sensorineural deafness, hypothyroidism, and frequent infections as well as the identification of a heterozygous *de novo* mutation in *CDC42*, i.e., p.Tyr64Cys.

## Introduction

Recently, we reported two unrelated individuals with thrombocytopenia accompanied by intellectual disability and a *de novo* mutation in *CDC42*, a critical molecule in the regulation of the cell cycle and the formation of the actin cytoskeleton^1,2^. Both patients shared intellectual disability, distinctive facial features, macrothrombocytopenia (increased platelet size and decreased platelet count), camptodactyly, and structural brain abnormalities with sensorineural hearing loss. Through whole exome analyses, we identified the exact same *de novo* mutation in *CDC42*, i.e., p.Tyr64Cys, in both patients. The characteristic hematologic feature, i.e., macrothrombocytopenia, was compatible with existing evidence of a similar platelet phenotype in a model organism (*Cdc42* homozygous knockout mice)^3^. The identification of multiple unrelated patients with similar phenotypes and exactly the same *de novo CDC42* mutation enabled us to confirm that these patients represent a new syndromic form of thrombocytopenia, which was eponymized as “Takenouchi-Kosaki syndrome” (OMIM #616737). Later, Motokawa *et al*. reported a third patient with this condition^4^, and we recently evaluated a fourth patient.

The cardinal features of Takenouchi-Kosaki syndrome include intellectual disability accompanied by macrothrombocytopenia. However, the detailed clinical characteristics and mechanistic basis of the macrothrombocytopenia observed in these individuals remain to be elucidated. In the present study, we sought to delineate the pathogenetic basis of the macrothrombocytopenia observed in Takenouchi-Kosaki syndrome and performed a functional analysis of p.Tyr64Cys in *cdc*-*42* using CRISPR/Cas9-mediated gene editing and *Caenorhabditis elegans*.

## Results

### Phenotypic spectrum of Takenouchi-Kosaki syndrome

A total of five patients with macrothrombocytopenia, intellectual disability, and a *de novo* heterozygous mutation in *CDC42*, i.e., p.Tyr64Cys, have been identified. Four of these patients were reported previously^1,2,4,5^. We have summarized the clinical characteristics of these five patients with Takenouchi-Kosaki syndrome in a tabular form (Table 1). The shared features among the five patients were as follows: macrothrombocytopenia (present in 5/5 cases), intellectual disability (5/5), structural brain abnormalities with sensorineural deafness (5/5), camptodactyly (4/5), hypothyroidism (3/5), frequent infections (3/5), and lymph edema (2/5). All five individuals exhibited macrothrombocytopenia; however, none of the five patients exhibited bleeding diathesis. All five patients had moderate to severe intellectual disability. Structural brain abnormalities and sensorineural deafness were present in all five patients who underwent neuroimaging studies. The patterns of brain abnormalities were rather nonspecific, with various degrees of ventriculomegaly and cerebellar atrophy. Camptodactyly was present in four female patients who were all in their teens or older. Hypothyroidism was present in three patients. Three patients had a history of recurrent bacterial infections: patient 1 had a history of fulminant streptococcus infection, and patient 3 had recurrent upper respiratory infections. Four patients (patients 1, 2, 3, and 4) had decreased counts of CD19-positive cells. Abnormalities in the lymphatic system were noted in two patients: patients 1 and 2 had lymph edema in their lower extremities, and patient 2 had protein-losing enteropathy.

**Table 1:** Clinical features in patients with Takenouchi-Kosaki syndrome.

Patient #	1	2	3	4	5	Frequency
Age/Sex	18 y/female	22 y/female	12 y/female	15 y/female	4 y/male	
Macrothrombocytopenia	Present	Present	Present	Present	Present	5/5
Intellectual disability	Present	Present	Present	Present	Present	5/5
Structural brain abnormalities and sensorineural deafness	Present	Present	Present	Present	Present	5/5
Camptodactyly	Present	Present	Present	Present	Absent	4/5
Frequent infections	Present	Absent	Present	Present	Absent	3/5
Hypothyroidism	Absent	Absent	Present	Present	Present	3/5
Lymph edema	Present	Present	Absent	Absent	Absent	2/5
Heterozygous *de novo* mutation in *CDC42*	p.Tyr64Cys	p.Tyr64Cys	p.Tyr64Cys	p.Tyr64Cys	p.Tyr64Cys	
References	Takenouchi *et al*.^1^	Takenouchi *et al*.^2^	Motokawa *et al*.^4^	Martinelli *et al*.^5^	Unpublished data	

### Genotype

All five patients had a single *de novo* amino acid substitution change in *CDC42*, i.e., c.191A>G p.Tyr64Cys. The details of the genetic analyses performed for patients 1–4 have been published elsewhere^1,2,4,5^. An exome analysis was performed for patient 5 as part of routine clinical practice, and this patient is not of Japanese descent. The p.Tyr64 is located within the switch II domain of *CDC42*^6^. An *in silico* analysis of p.Tyr64Cys in *CDC42* showed that this amino acid residue is highly conserved among species, with a high Combined Annotation Dependent Depletion score^7^ of 23.4 (phred). According to the list of post-transcriptional modification sites^8^, p.Tyr64 in *CDC42* is a target site of phosphorylation. According to recently proposed criteria for pathogenicity, the variant was interpreted as “pathogenic”^9^. p.Tyr64Cys was absent in the largest database of 2,049 normal Japanese individuals determined using whole-genome sequencing (Japanese Genome Variation Database)^10^, the exome sequencing of 1,208 normal Japanese individuals (Human Genetic Variation Database)^11^, and the 1,000 Genome^12^.

### Electron microscopy investigation of platelets in patients

**Figure 1:**
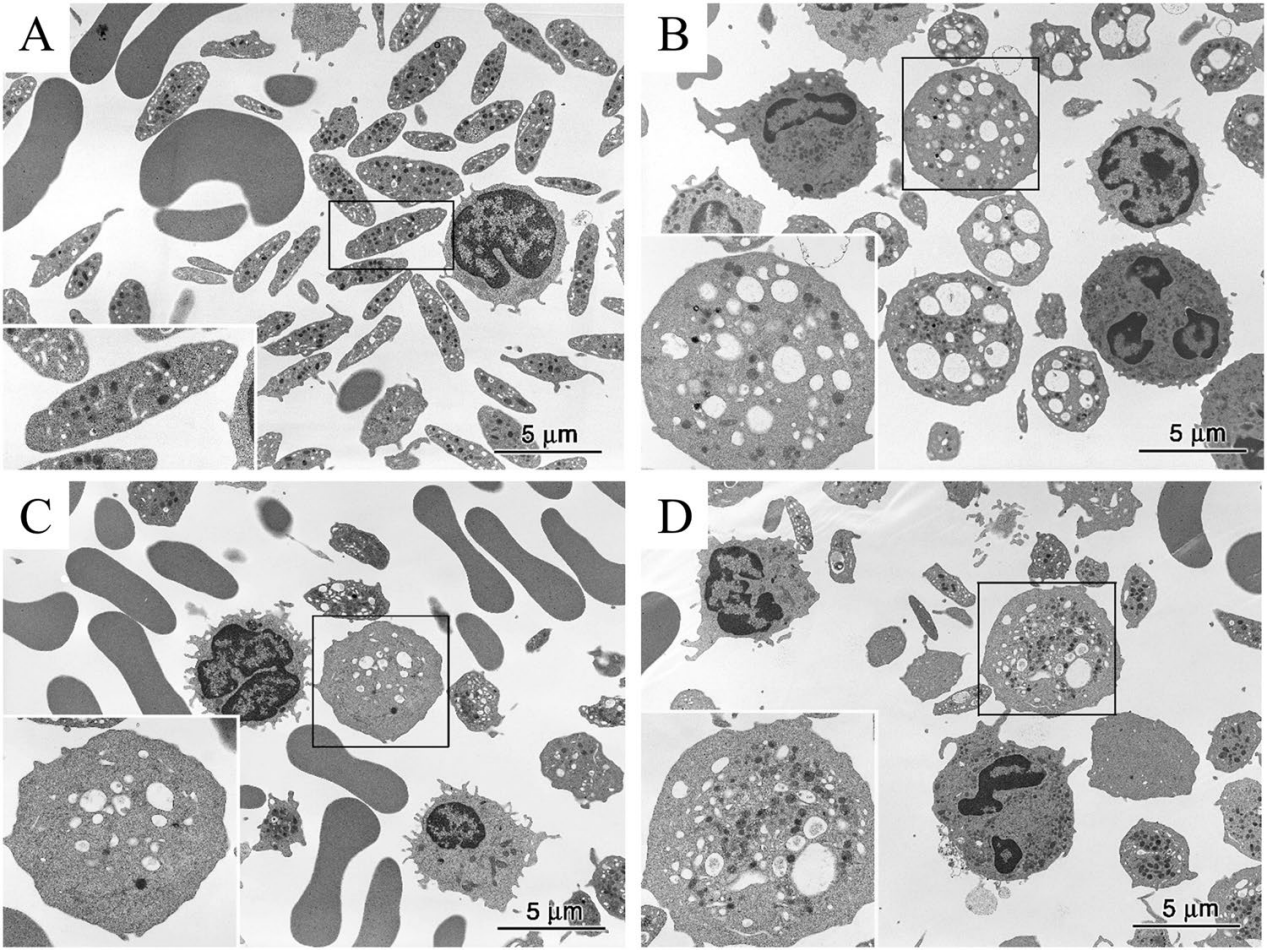
Electron micrographs of platelets from normal subjects and three patients. (**A**) Normal platelets have a disk-like appearance with a diameter of approximately 3.5 μm. The inset shows an enlarged platelet in the boxed region in which platelet granules and an open canalicular system are visible. (**B**–**D**) Some typical platelets of three patients are larger and spherical in shape. In these platelets, the number of α-granules is slightly decreased and the number of vacuoles with a diameter of approximately 0.5 μm and that are notably different from those belonging to the open canalicular system is increased. The insets show enlarged images of the boxed regions.

**Figure 2:**
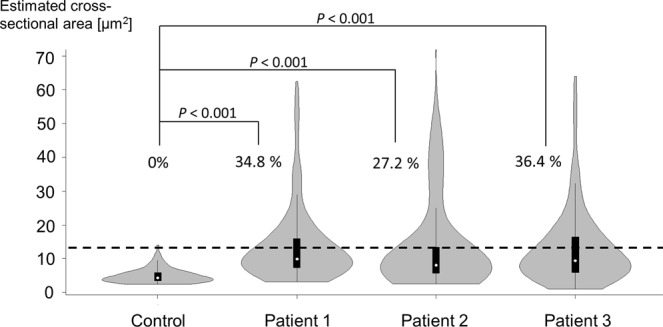
Skewed distribution of platelet size in patients. The vertical axis represents the estimated cross-sectional area [μm^2^]. The horizontal dotted line represents the cut-off value (12.6 μm^2^) of the estimated cross-sectional area for macrothrombocytes. Note that the distributions of platelet size were skewed positively (upwards) in patients 1–3. Macrothrombocytes comprised none (1/120) of the platelets in the control, 34.8% (47/135) of the platelets in patient 1, 27.2% (24/88) of the platelets in patient 2, and 36.4% (51/140) of the platelets in patient 3.

**Figure 3:**
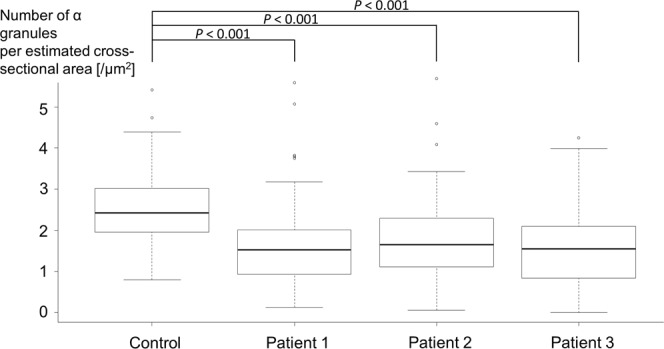
Decreased α-granule density in patients’ platelets. The vertical axis represents the number of α-granules per estimated cross-sectional area [μm^2^]. Note that the α-granule density is significantly lower in all three patients than in the control.

In healthy controls, platelets are smaller than red blood cells and white blood cells and have a disk-shaped appearance and a diameter of long and short axes of 3.5 ± 0.7 μm and 1.4 ± 0.5 μm, respectively (Fig. 1A). α-Granules, dense granules, and mitochondria were abundant in the cytoplasm of the platelets, and microtubules are present at the marginal ends^13^. Structures that appear to be small vacuoles in normal platelets were actually channels of the open canalicular system derived from the invagination of cell membranes. In the three patients with Takenouchi-Kosaki syndrome, electron microscopic images showed the presence of platelets that were larger in size and had a spherical shape (Fig. 1B–D). A violin plot of the estimated cross-sectional area of the platelets showed a positive skew, indicating that a substantial proportion of the platelets in patients 1–3 could be regarded as macrothrombocytes, although a large proportion of the platelets remained relatively unchanged in size (Fig. 2). By counting the numbers of macrothrombocytes (platelets with a cross-sectional area exceeding 12.6 μm^2^ and having a spherical shape), the ratio of macrothrombocytes to normal-sized platelets was shown to be significantly higher in patients 1–3, compared with the control (*P* < 0.001, Fisher’s exact test). In terms of platelet morphology, long and short axes of platelets from the three patients were 3.9 ± 1.1 × 3.2 ± 1.3 μm, 4.1 ± 1.5 × 2.9 ± 1.6 μm, 4.1 ± 1.9 × 2.8 ± 1.4 μm, respectively. The ratio of normal discoid shaped platelets was significantly decreased in patients 1–3 (*P* < 0.001, Fisher’s exact test) (Table 2). As for the organelles within the platelets, the number of α-granules was slightly reduced and the number of vacuoles with a diameter of approximately 0.5 μm or more and that were clearly different from those of the open canalicular system was increased. Consistent with these observations, our quantitative measurements showed that the number of α-granules per estimated cross-sectional area was decreased in patients (*P* < 0.001, Dunnett’s test) (Fig. 3). The large platelets with α-granules depletion were reminiscent of those in patients with Gray platelet syndrome^14^. Large vacuoles were particularly evident in platelets from patient 1 and appeared to be increased in number in patients 2 and 3; however, the numbers per area were to too small for statistical analysis. In platelets from three of the patients, interspersed microtubules were observed.

**Table 2:** Platelet shape in the control and the three patients.

	Ratio of normal discoid-shaped platelets	*P* value
Control	93% (111/120)	—
Patient 1	20% (27/135)	<0.001
Patient 2	34% (30/88)	<0.001
Patient 3	34% (48/140)	<0.001

### Functional analysis using *C*. *elegans*

We generated a model organism of *cdc*-*42* p.Tyr64Cys using *C*. *elegans* for the following reasons: *C*. *elegans* can be grown less expensively and can be frozen and then thawed and revived when needed. Along with its fully mapped genome and extensively studied cell fate specification, many gene-editing and analytic methods have been established for *C*. *elegans* and are commonly available. *C*. *elegans* also has an ortholog of human *CDC42*, namely *cdc*-*42*, and the nucleotide sequence in the vicinity of the p.Tyr64Cys mutation is highly conserved between *CDC42* in humans and *cdc*-*42* in *C*. *elegans*. Using the CRISPR/Cas9-mediated gene editing methods^15^, we successfully generated worms carrying one or two alleles of the missense mutation, [*cdc*-*42*^p.Tyr64Cys/+^] and [*cdc*-*42*^p.Tyr64Cys^], i.e., *tm9602*/+ strain and *tm9602* strain respectively, from the standard N2 background.

### Validation of knock-in model generated through gene editing

We first validated the knock-in model by confirming that a protruding vulva and mild morphological changes in gonads were observed in approximately 30% of all the knock-in worms homozygous for the p.Tyr64Cys mutant allele and in approximately 10% of the worms that were heterozygous for the p.Tyr64Cys mutant allele (data not shown). These morphological changes were compatible with reduction-of-function of *cdc*-*42* rather than the loss-of-function of *cdc*-*42* in prior reports^16–18^.

### Analysis of apoptotic cell clearance in gonads and embryos

**Figure 4:**
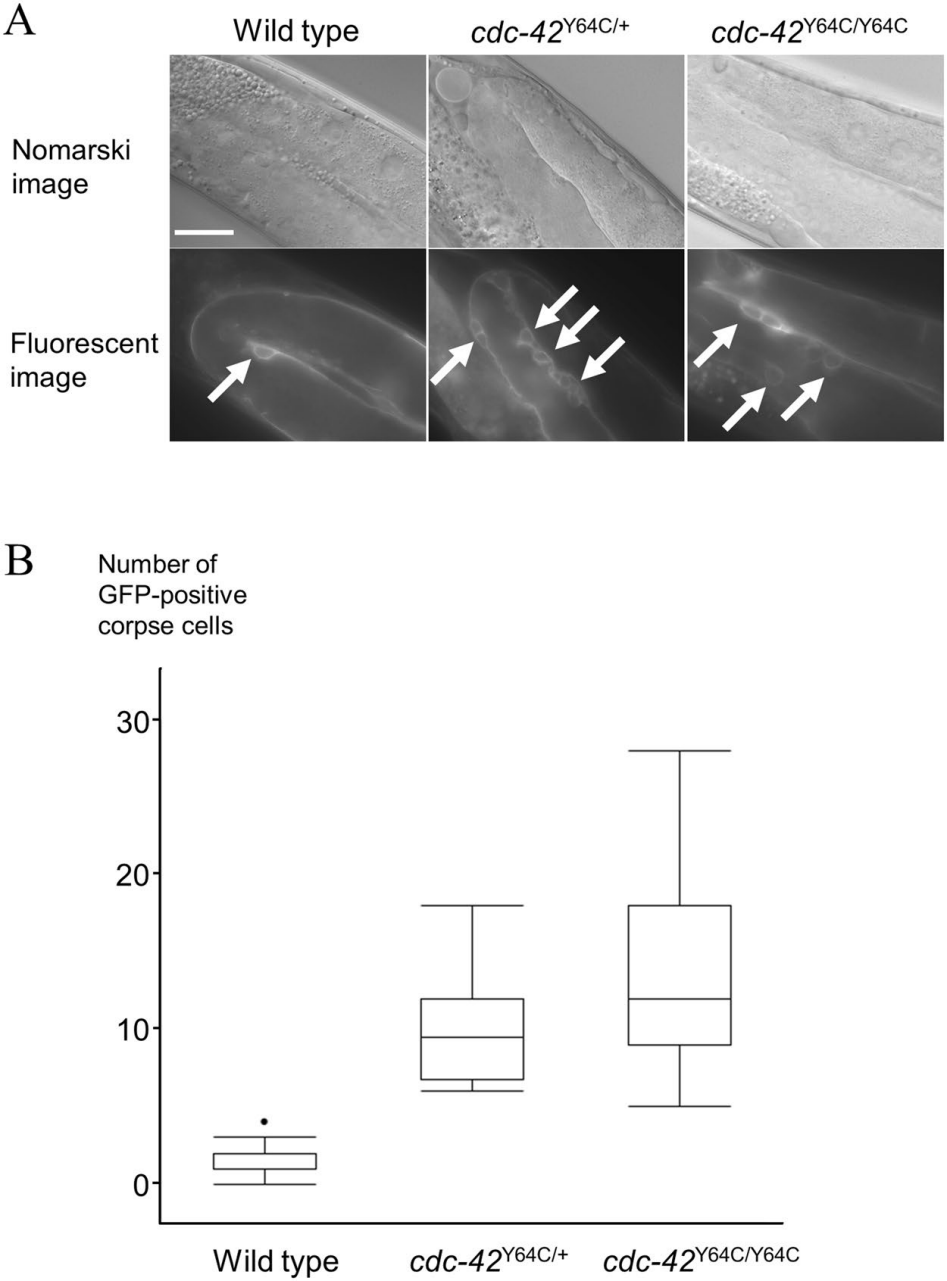
p.Tyr64Cys mutation of CDC-42 affects apoptotic cell clearance in gonads. (**A**) Nomarski and fluorescent images (x100) of gonads in L4-stage hermaphrodites in a wild type worm (left), a knock-in model of heterozygous p.Tyr64Cys-mutated *cdc*-*42* (*cdc*-*42*^Y64C/+^, middle), and a knock-in model of homozygous p.Tyr64Cys-mutated *cdc*-*42* (*cdc*-*42*^Y64C/Y64C^, right). The white arrows indicate GFP-positive corpse cells. Scale bar = 20 µm. (**B**) Bar chart showing the average number of corpse cells expressing GFP in wild type, *cdc*-*42*^Y64C/+^, and *cdc*-*42*^Y64C/Y64C^ L4-stage hermaphrodites (n = 15–36). The error bars indicate the standard error of the mean.

We opted to analyze the number of corpse cells as a functional consequence of mutant *cdc*-*42* p.Tyr64Cys because the involvement and roles of *cdc*-*42* in the process of apoptosis have been well established in previous studies of *C*. *elegans*, and corpse cells in gonads and eggs can be readily visualized and are countable under microscopy. The numbers of corpse cells derived from gonadal cells in the gonads were increased in heterozygous (*tm9602*/+) and homozygous (*tm9602*) worms in a gene dosage-dependent manner (*P* < 0.05, nonparametric max3 test) (Fig. 4). Furthermore, the number of corpse cells derived from embryonic cells was also increased in the homozygous (*tm9602*) strain, compared with wild-type worms (*P* < 0.0001, Student’s *t*-test). Hence, this increase in the number of corpse cells was ascribed to the decreased clearance of apoptotic cells in *cdc*-*42* knockout worms^19^.

### RNA interference

**Figure 5:**
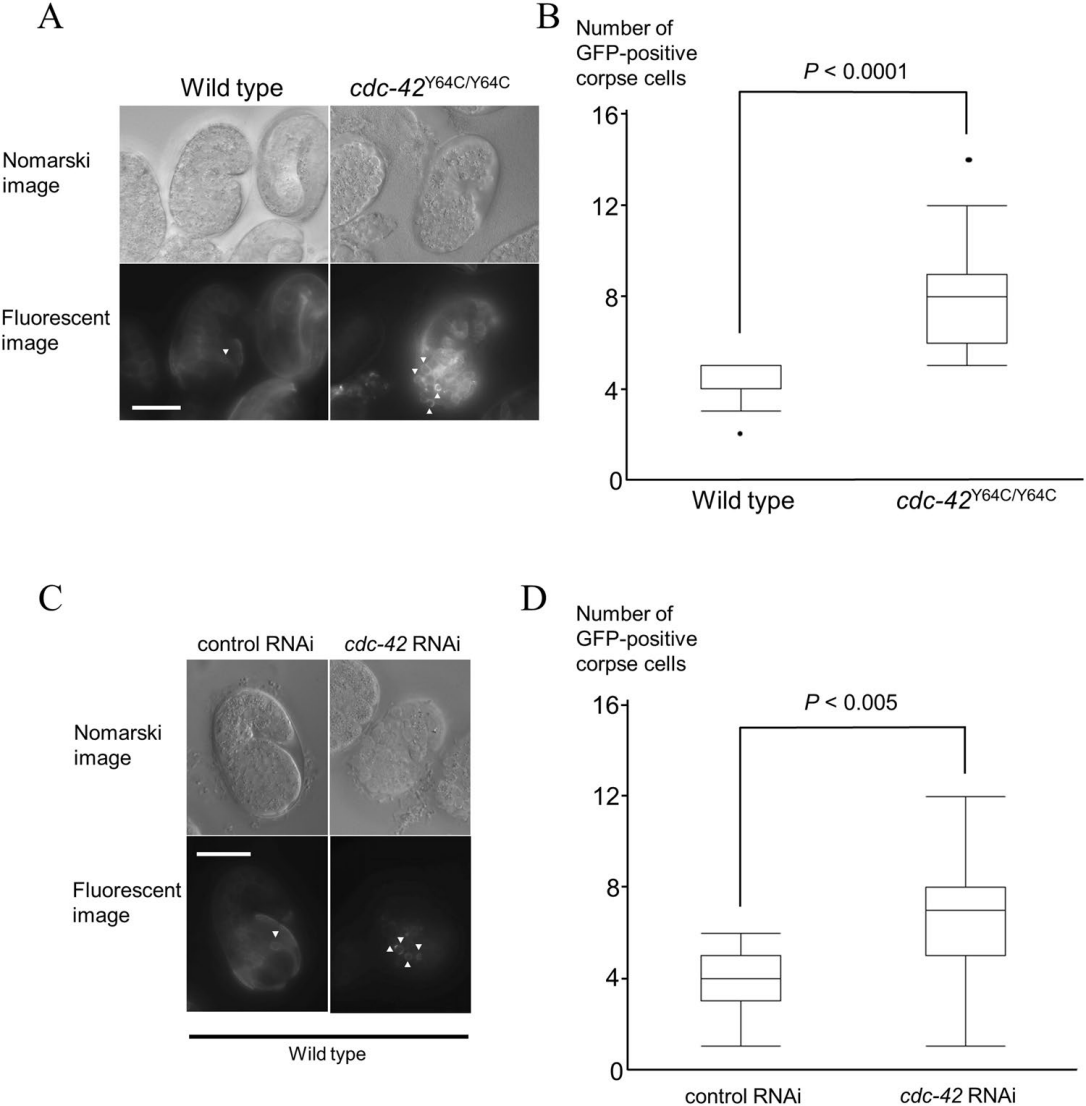
Both the p.Tyr64Cys mutation of CDC-42 and *cdc*-*42*-specific RNA interference affect apoptotic cell clearance in embryonic cells. (**A**) Nomarski and fluorescent images (x100) of comma-stage eggs in a wild type (left) and a knock-in model of homozygous p.Tyr64Cys-mutated *cdc*-*42* (*cdc*-*42*^Y64C/Y64C^; right). The white arrow heads indicate GFP-positive corpse cells. (**B**) Bar chart showing the average number of corpse cells expressing GFP in wild type and *cdc*-*42*^Y64C/Y64C^ comma-stage eggs (n = 12–36). (**C**) Nomarski and fluorescent images (x100) of comma-stage eggs treated with empty-vector (L4440) RNAi (left) and *cdc*-*42*-specific RNAi (right). The white arrows indicate GFP-positive corpse cells. (**D**) Bar chart showing the average number of corpse cells expressing GFP in comma-stage eggs treated with L4440 (RNAi) and *cdc*-*42* (RNAi) (n = 12–22). The error bars indicate the standard error of the mean. Scale bar = 20 µm.

To distinguish between the dominant negative mechanism and the hypomorphic mechanism, we analyzed the numbers of corpse cells in *cdc*-*42* knockdown worms mediated by a *cdc*-*42*-specific RNAi clone. The application of a *cdc*-*42*-specific RNAi clone resulted in an increased number of corpse cells (*P* < 0.005, Student’s *t*-test), similar to the *tm9602* strains (Fig. 5).

## Discussion

In the present report, we showed that Takenouchi-Kosaki syndrome is a clinically recognizable condition characterized by a combination of macrothrombocytopenia, intellectual disability, sensorineural hearing loss with structural brain abnormalities, camptodactyly, and frequent infections and decreased CD19-positive cells. An electron microscopy investigation of platelets demonstrated a markedly increased size of platelets accompanied by an increase in vacuoles. We then successfully generated a knock-in model of *cdc*-*42* mutated *C*. *elegans* carrying this p.Tyr64Cys mutation through CRISPR/Cas9-mediated gene editing. The knock-in model exhibited delays in the clearance of corpse cells both in the gonads and embryonic cells, and these findings were reproduced using RNAi in a gene dosage-dependent fashion. The present results suggest that the mechanistic basis of macrothrombocytopenia in Takenouchi-Kosaki syndrome involves a hypomorphic effect exerted by the p.Tyr64Cys mutation in *CDC42*.

Following our initial observation of two patients with Takenouchi-Kosaki syndrome, Martinelli *et al*. recently reported a series of patients with *de novo* mutations in *CDC42* and various phenotypes. In their report, one patient (subject #3) had the p.Tyr64Cys mutation in *CDC42* and exhibited macrothrombocytopenia, intellectual disability, hypothyroidism, camptodactyly, and recurrent infections^5^, all of which were all compatible with the phenotypes of our previously reported patients. Their functional analysis showed that the p.Tyr64Cys mutation was associated with the largest reduction, by far, in GTPase activity in an *in vitro* assay; this finding is compatible with the notion that patients with p.Tyr64Cys represent the most prototypic and severe end of the clinical spectrum.

The characteristic platelet phenotype described in this report provides a specific diagnostic clue to Takenouchi-Kosaki syndrome. Despite the presence of giant platelets, none of the patients exhibited a clinically overt bleeding tendency. The electron microscopy findings for the platelets from the presently reported patients, such as the increased number of vacuoles, were compatible with those obtained in double knockout mice (*Cdc42* and *Rac1*)^20^. We would like to emphasize that macrothrombocytopenia, which is readily detectable during routine clinical laboratory examinations, is a useful diagnostic clue in the differential diagnosis of patients with intellectual disabilities. From the perspective of future research directions, further functional and morphological analyses of platelets, i.e., expression analysis of surface markers, proplatelet formation assays and analyses of tubulin organization and action cytoskeleton of platelets in comparison to prior studies^3,20–22^, would provide further insight into the mechanistic basis of macrothrombocytopenia in Takenouchi-Kosaki syndrome.

We showed that the mechanistic basis of Takenouchi-Kosaki syndrome is likely to involve a hypomorphic effect derived from the mutant allele. Through an *in vivo* analysis of mutant *C*. *elegans*, we observed the abnormal clearance of corpse cells using two complementary methods and confirmed that the mutant allele is hypomorphic. First, we assessed the clearance of corpse cells in the gonads, which occurs as a physiological apoptotic process. The number of corpse cells in both the mutant worms and the null-mutant worms increased in a gene dose-dependent manner. This observation was compatible with the notion that p.Tyr64Cys represents a hypomorphic allele. Second, we evaluated the clearance of corpse cells in worms during the embryonic stage. The self-fertilization of *C*. *elegans* results in diploid embryos. Embryos at the comma stage exhibit corpse cell clearance as a physiological apoptotic process. Similar to the findings for the gonads, an increased number of corpse cells was observed at this stage of worm development. Furthermore, the increase in the number of corpse cells was reproduced in the gonads of worms with one mutated allele. The increase in the number of corpse cells was reproduced using RNAi. Given that RNAi specifically suppresses the expression of *cdc*-*42*, this reproducibility using RNAi was compatible with the notion that p.Tyr64Cys represents a hypomorphic allele, rather than a neomorphic or antimorphic allele.

In conclusion, Takenouchi-Kosaki syndrome is a clinically recognizable human disease that is characterized by macrothrombocytopenia, intellectual disability, sensorineural hearing loss with structural brain abnormalities, camptodactyly, and frequent infections as well as a *de novo* heterozygous single amino acid substitution change in *CDC42*, i.e., p.Tyr64Cys. The p.Tyr64Cys mutation in *CDC42* exerts a hypomorphic effect and represents the most severe and prototypic end of the *CDC42* spectrum. In patients with intellectual disability, the identification of giant platelets with an increased number of vacuoles in routine laboratory testing is a diagnostic clue for Takenouchi-Kosaki syndrome.

## Materials and Methods

### Study subjects

Individuals with thrombocytopenia, intellectual disability, and a p.Tyr64Cys mutation in *CDC42* were identified through a MEDLINE search and our local collaborative research network.

### Platelet preparation and electron microscopy

The research protocol was approved by Keio University School of Medicine Ethics Committee. All methods were carried out in accordance with the World Medical Association Declaration of Helsinki. After obtaining informed consent, venous blood samples from patients 1, 2, and 3 were directly collected into respective syringes containing 3.8% sodium citrate as an anticoagulant (9:1 whole blood:anticoagulant, v/v). At each collection time, blood was also sampled from healthy 3 normal volunteers who had not received any medication for at least 10 days. The tubes containing the blood samples were allowed to settle for 2 hours at room temperature to obtain platelet-rich plasma (PRP). PRP was carefully harvested, and prostaglandin E1 (PGE1, 1 μM; Sigma-Aldrich) was added to the PRP. Platelets were sedimented by centrifugation at 900 *g* for 15 min and resuspended in HEPES-Tyrode’s washing solution (pH 7.4) containing 137 mM NaCl, 2.7 mM KCl, 0.4 mM NaH2PO_4_, 12 mM NaHCO_3_, 1 mM MgCl_2_, 5 mM HEPES, 1 μM PGE1, 0.35% bovine serum albumin (BSA), and 0.1% glucose. The suspension was kept at 37 °C for 30 min. After centrifugation at 900 *g* for 10 min, platelets were finally suspended in the above-mentioned HEPES-Tyrode’s solution without PGE1 and maintained at 37 °C prior to use.

Washed platelets obtained from control and 3 patients were fixed by mixing with an equal volume of 2% glutaraldehyde in 0.1 M phosphate buffer (pH 7.4) for 30 min. The fixed cells were transferred to Eppendorf tubes, then centrifuged at 3000 rpm for 3 min at 4 °C. The platelet pellets were washed 3 times in 0.1 M phosphate buffer, post-fixed with 1% osmium tetroxide for 1 hour at 4 °C, dehydrated with a graded ethanol series, and embedded in Epon 812 according to conventional methods. Thin sections were cut with a diamond knife and stained with uranyl acetate and lead citrate, then examined using a JEM-1400plus electron microscope (JEOL, Tokyo, Japan) at an accelerating voltage of 80 kV.

### Quantitative measurement of size and microstructure of platelets

Using electron microscopic images, we performed quantitative measurements of the long and short axes of the platelets and counted the numbers of α-granules and vacuoles; we then compared the results of patients 1–3 with those of the control. The cross-sectional area of the platelets was estimated using the mathematical formula for the area of an ellipse, i.e., $S=\pi {ab}$, where *a* represents the semi-major axis and *b* represents the semi-minor axis. The typical size of normal platelets ranges from 3 to 4 μm. A diameter of 4 μm corresponds to a circular area of 3.14 × 2 × 2 = 12.6 μm^2^. Therefore, we defined macrothrombocytes as platelets with a cross-sectional area exceeding 12.6 μm^2^. Vacuoles were counted only when their diameter exceeded 0.5 μm.

### Strains of *Caenorhabditis elegans*

All strains of *C*. *elegans* were seeded with *Escherichia coli* OP50. All the experiments were performed at 20 °C using standard techniques^23^. The wild-type strain Bristol N2 was obtained from the Caenorhabditis Genetics Center (CGC, Minneapolis, MN). Strains carrying the following mutations were obtained from the trimethylpsoralen/ultraviolet-mutagenized library, as described previously^24^.

### Generation of knock-in model of *C*. *elegans* using CRISPR/Cas9-mediated gene editing

We chose to generate a knock-in model, rather than a transgenic model, of the p.Tyr64Cys mutation in *C*. *elegans* using the CRISPR/Cas9 system and one copy of the abnormal allele and one copy of the normal allele to better recapitulate the pathogenesis in humans with Takenouchi-Kosaki syndrome. A comparison of the human CDC42 protein (NP_001782.1) sequence with that for *C*. *elegans* CDC-42^25^ revealed a high degree of evolutionary conservation of the protein sequence flanking the p.Tyr64Cys missense mutation in humans^5^, and the p.Tyr64Cys corresponded to p.Tyr64Cys in *C*. *elegans*. To generate the strain with the *cdc*-*42* missense mutation, *tm9602*, we used the CRISPR/Cas9 method as described previously^15^. First, we selected two spots of the guide sequence for *cdc*-*42* to be used for Cas9 cleavage (#1_AGGTCACAGTAATGATCGG and #2_TTTCTTGTTTGCTTCTCCG) through “CRISPR Design” (http://crispr.mit.edu). The two sequences were inserted into a Cas9-sgRNA (single guide RNA) expression vector (*pPD162*) using Addgene^26^. The primers used to generate the sgRNAs according to the infusion method were as follows: sgRNA_#1, 5′-ACC TCC TAT TGC GAG ATG TCT TGA GGT CAC AGT AA-3′, 5′-TCT AGC TCT AAA ACC CGA TCA TTA CTG TGA CCT CAA-3′; and sgRNA_#2, 5′-TCC TAT TGC GAG ATG TCT TGT TTC TTG TTT GCT TCT-3′, 5′-GCT ATT TCT AGC TCT AAA ACC GGA GAA GCA AAC AAG AA-3′. Next, to generate the *cdc*-*42* genome fragment plasmid (i.e., the targeting vector), *pPD95*.*79*_ *cdc*-*42*^p.Tyr64Cys^, a mutated *cdc*-*42* genome, was amplified by PCR and sewing PCR using the following primers: #1; 5′-GAA ATG AAA TAA GCT TTC TCT GCG TAT CTC ACC AC-3′, 5′-GAT CAT TAC TGT CAC GGC GT-3′; #2; 5′-TAC GCC GTG ACA GTA ATG ATC G-3′, 5′-GGA GAA GCA AAC AAG GAA CA-3′, #3; 5′-ACC GAC GTG TTC CTT GTT TGC T-3′, 5′-CCA ATC CCG GGG ATC GAA ATT CTA TAC GAA ACA AT-3′. The fragment was inserted into the *pPD95*.*79* plasmid using the EcoRI and BamHI sites. The primers used to generate the target vector according to infusion methods were as follows: 5′-TTC AGG TGA CAG TAA TGA TCG-3′, and 5′-GCC ACC GAT CAT TAC TGT CA-3′. To generate integration lines with *cdc*-*42*^p.Tyr64Cys^, two sgRNAs and one target vector were injected using standard *C*. *elegans* microinjection methods^27^ with the *myo*-*2::Venus* selection marker. Strain *tm9602* was backcrossed twice with N2. To generate a worm strain carrying one allele of the missense mutation, we balanced *tm9602* worms with *mIn1* [*mIs14dpy*-*10* (*e128*)].

### Analysis of corpse cells

Since CDC-42 plays a critical role in apoptotic cell clearance^19,28^, we evaluated the functional state of *cdc*-*42*^p.Tyr64Cys^ by counting corpse cells, which reflect the clearance of apoptotic cells, in both somatic cells (gonads) and embryonic cells (comma-stage eggs). Functional abnormalities in the *tm9602* strain (homozygous mutant), the *tm9602*/+ strain (heterozygous mutant), and *cdc*-*42* knockdown worms were studied with regard to the clearance of post-apoptotic cells by engulfment cells. The increases in corpse cell numbers derived from both gonadal cells and embryonic cells were interpreted as a quantitative measurement of the clearance function of post-apoptotic cells. To assay the number of corpse cells, we used the integrated array *SmIs34*, which contains *ced*-*1p::ced*-*1::gfp*. The transgenic strain was obtained through the Caenorhabditis Genetics Center.

### RNA interference

To generate *cdc*-*42* knockdown worms, we used RNA interference (RNAi) methods. RNAi was performed by feeding animals dsRNA-producing bacteria, as described previously^29^. Briefly, the RNAi clones were transformed into *E*. *coli* HT115(DE3); then, approximately 10–20 P0 animals at the early L1 stage were transferred to plates containing RNAi-bacteria grown on 100 µg/mL of ampicillin and 1 mmol/L of isopropyl-beta-D-thiogalactopyranoside (IPTG). To analyze the phenotypes of *cdc*-*42*-knockdown worms, the numbers of apoptotic corpse cells were counted in both the P0 generation at the L4 larval stage and the F1 generation at the comma-stage. We then compared the numbers of corpse cells in *cdc*-*42*-knockdown and control worms. The RNAi clone was obtained from the Ahringer RNAi library.

### Microscope

Differential interference contrast and fluorescence images were obtained using a BX51 microscope equipped with a DP30BW CCD camera (Olympus Optical Co., Ltd, Tokyo, Japan).

### Statistical analyses

The results are expressed as mean ± standard deviation. All the analyses were performed using R software, version 3.4.3. The Dunnett’s test was used to analyze the numbers of granules per estimated cross-sectional area. The Fisher’s exact test was used to analyze the ratio of macrothrombocytes to normal-sized platelets. Nonparametric max 3 (a procedure for testing the association between a biallelic single nucleotide polymorphism and a quantitative trait using the maximum value of the three nonparametric trend tests derived for the recessive, additive, and dominant models) was used to analyze the gene dosage effect on the number of corpse cells in gonads^30^. The Student’s *t*-test was used to compare the numbers of corpse cells in embryonic cells and the effect of RNAi. Differences with a *P* value of 0.05 were considered to indicate a statistical significance.
